# Improved Outcomes in Eosinophilic Esophagitis with Higher Medication Possession Ratio

**DOI:** 10.3390/medicines11040008

**Published:** 2024-03-26

**Authors:** Nathan T. Kolasinski, Eric A. Pasman, Cade M. Nylund, Patrick T. Reeves, Daniel I. Brooks, Katerina G. Lescouflair, Steve B. Min

**Affiliations:** 1 Department of Pediatrics, Walter Reed National Military Medical Center, Bethesda, MD 20889, USA; 2 Department of Pediatrics, Uniformed Services University, Bethesda, MD 20889, USA; 3 Department of Pediatrics, Naval Medical Center San Diego, San Diego, CA 92134, USA; 4 Department of Research Programs, Walter Reed National Military Medical Center, Bethesda, MD 20889, USA

**Keywords:** eosinophilic esophagitis, medication possession ratio, compliance, adherence, cost, safety

## Abstract

Eosinophilic esophagitis (EoE) disease activity can be caused by treatment non-adherence. Medication possession ratio (MPR) is an established metric of medication adherence. A higher MPR correlates with better outcomes in several chronic diseases, but MPR has not been investigated with respect to EoE. A retrospective cohort study was performed using an established EoE registry for the years 2005 to 2020. Treatment periods were identified, MPRs were calculated, and medical records were assessed for histologic remission (<15 eos/hpf), dysphagia, food impaction, stricture occurrence, and esophageal dilation that corresponded to each treatment period. In total, 275 treatment periods were included for analysis. The MPR in the histologic remission treatment period group was 0.91 (IQR 0.63–1) vs. 0.63 (IQR 0.31–0.95) for the non-remission treatment period group (*p* < 0.001). The optimal MPR cut-point for histologic remission was 0.7 (Sen 0.66, Spec 0.62, AUC 0.63). With MPRs ≥ 0.7, there were significantly increased odds of histologic remission (odds ratio 3.05, 95% confidence interval 1.79–5.30) and significantly decreased odds of dysphagia (OR 0.27, 95% CI 0.15–0.45), food impaction (OR 0.26, 95% CI 0.11–0.55), stricture occurrence (OR 0.52 95% CI 0.29–0.92), and esophageal dilation (OR 0.29, 95% CI 0.15–0.54). Assessing MPR before repeating an esophagogastroduodenoscopy may decrease unnecessary procedures in the clinical management of eosinophilic esophagitis.

## 1. Introduction

Eosinophilic esophagitis (EoE) is a chronic, immune-mediated cause of esophageal inflammation and dysfunction, with a prevalence of 0.5–1 cases per 1000 people [1,2]. It is a multifactorial disease that arises from a complex interplay of genetic, environmental, and immunologic factors which result in eosinophilic infiltration of the esophagus [3,4]. Classic clinical symptoms of EoE include feeding intolerance, dysphagia, odynophagia, and food impaction. Other presenting symptoms may also include heart burn, chest pain, regurgitation, and abdominal pain [1,5,6]. Although the presentation of EoE varies among different age groups, the diagnostic criteria are the same in children and adults [4]. The diagnosis of EoE requires esophagogastroduodenoscopy (EGD) with biopsy, demonstrating 15 or greater eosinophils per high-power field (eos/hpf). If EoE remains untreated, severe complications such as esophageal stricture may develop, potentially necessitating esophageal dilation [1,3,5].

Common, first-line medications used to treat EoE include proton-pump inhibitors (PPIs) and topical steroids (TSs). PPIs are effective for histologic remission in 50 percent of patients with EoE [7]. TS use in EoE is more effective than the use of PPIs, but is largely limited to oral viscous budesonide and swallowed fluticasone [1,5]. Very recently, biologic medications able to treat EoE, such as Dupilimab, have been approved by the United States Food and Drug Administration [8,9,10]. Elimination diets have also been used with success to treat EoE [1,5,10]. While variability in the practice of EoE management among gastroenterologists has been well documented, PPIs and TSs remain the most-common recommendations for first-line treatment, respectively [11]. While treatment approaches are similar in the pediatric population, patient age and the minimization of side effects must be especially considered [4].

When embarking on medical therapy for EoE with any class of medication, adherence to a prescribed medication treatment plan is paramount for the successful pharmacologic treatment of EoE [6,12,13]. Additionally, regardless of the treatment modality (medical or dietary), managing EoE typically involves performing EGDs with biopsies to monitor for a response to treatment. These are typically performed 8–12 weeks after a change in therapy, and the biopsy results are critically important in guiding treatment adjustments, if indicated [5,7,14]. The over-arching goal of EoE treatment is to achieve symptom control, histologic remission, and the prevention of complications like strictures [3,6,7,13].

Histologic EoE disease activity may not correlate with clinical symptoms, but histologically active disease may still result in the progression of fibrosis and adverse outcomes such as esophageal strictures and food impactions [2]. Increasingly, evidence has shown that EoE maintenance therapy is needed to achieve histologic remission and halt the progression of fibrostenosis [13,15,16]. Further commentary has emphasized the need to consider therapy for EoE in the context of real-world practice as, currently, assessing and modifying treatment often necessitates monitoring EoE disease activity via repeat EGD(s) with biopsy [17]. There are cases of EoE that are refractory to treatment, which may necessitate the change or escalation of therapy, but non-adherence to a prescribed medication regimen could be a factor in patients being mistakenly labeled as having refractory disease.

Medication Possession Ratio (MPR) is an established corollary of adherence to medical treatment, with a higher MPR leading to better outcomes in several chronic diseases. MPR is typically defined as the number of days supplied with a medication divided by the number of days in a specific treatment period; it ranges from 0 to 1, where a value of 1 corresponds to the potential for 100% treatment adherence [18]. An MPR of ≥0.8 (i.e., possessing a medicine for 80% of the treatment period) has been regarded positively as “high” or “good” adherence [19,20]. MPR has been shown to correlate with disease control in AIDS, asthma, hyperlipidemia, osteoporosis, and type 2 diabetes, but it has not been investigated in EoE [19,20,21,22].

The aim of this study was to evaluate the association of MPR and the outcomes of histologic remission, dysphagia, food impaction, stricture occurrence, and esophageal dilation in adults with EoE. Preliminary data from this study was presented at national conferences in abstract form [23,24]. In contrast to what has previously been presented, this manuscript fully describes our study methods, comprehensively presents all our data, offers a robust discussion, and states our detailed conclusion.

## 2. Materials and Methods

**Figure 1:**
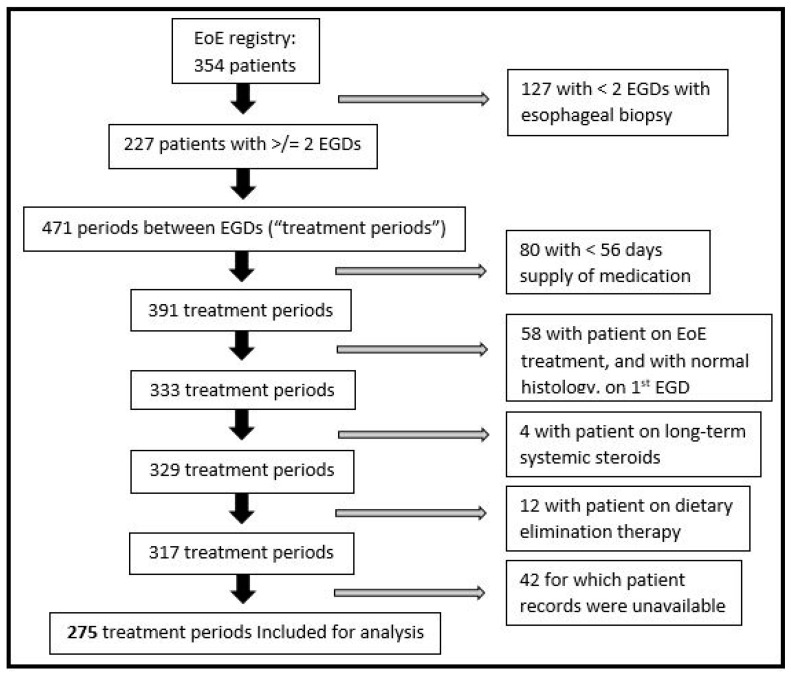
Inclusion and exclusion flowchart.

We conducted a retrospective cohort study in which an adult EoE registry at Walter Reed National Military Medical Center (WRNMMC) was reviewed from 2005 to 2020. Military Healthcare System (MHS) beneficiaries at WRNMMC receive all medical and surgical care, lab and pathology services, and pharmacy benefits free of cost; this enables the potential for great continuity of care in our patient population. An additional benefit for MHS providers is that medical, laboratory, and prescription records are housed on a single electronic medical record platform. The EoE registry at WRNMMC is composed of adults who, after they were diagnosed with EoE, consented to having their EoE-related medical data recorded by study investigators. Patients from the EoE registry were included for analysis in our study if they had an initial EGD that showed ≥15 eos/hpf on esophageal biopsy, were treated with any formulation of PPIs, TSs, or combination therapy (PPI + TS), and had a subsequent EGD performed at least 8 weeks after treatment initiation (Figure 1).

**Figure 2:**
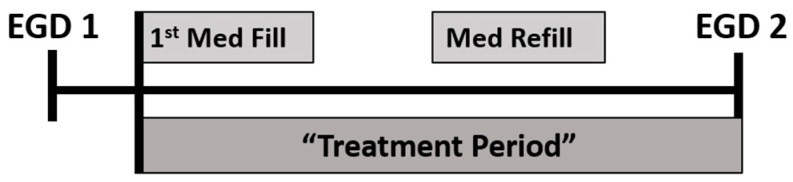
Treatment period determination.

The number of treatment periods, defined as any period between 2 EGDs in which a treatment for EoE was rendered, was then determined (Figure 2). As EoE is a chronic condition and patients with EoE may have multiple endoscopies over the course of their lives, each patient had the potential to generate multiple treatment periods for analysis. In the case of successive treatment periods corresponding to the same patient, the latter treatment period was excluded from analysis if the patient achieved histologic remission in the former treatment period. Treatment periods were excluded if the patient had a <56-day (<8 weeks) supply of medication, if the baseline EGD for the treatment period had normal histology, if the patient was on diet elimination therapy or systemic steroids, or if medical records were unavailable (Figure 1). A minimum 56-day (8 week) supply of medication was selected as the consensus viewpoint in that, if a patient with EoE is to achieve histologic remission on a given therapy, they will do so after ≥8 weeks of treatment [25]. Lastly, despite the robustness of the MHS medical record system, it does not extend in full to remote duty stations; thus, treatment periods during which active duty military personnel were receiving care while stationed abroad and/or in austere environments were not included for analysis, as their prescription data was not entirely verifiable.

**Figure 3:**
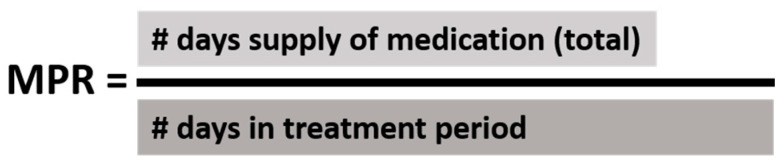
Medication possession ratio calculation.

MPR was calculated by determining the days of medication supply for each subject in each treatment period, and dividing that value by the number of days in between the first medication fill and the second EGD of the respective treatment period (Figure 3). The days of medication supply was obtained by the direct interrogation of electronic pharmacy records. In this manner, MPRs for PPIs, TSs, and combination therapy were determined for all included treatment periods. Medical records—including outpatient clinic and emergency department notes, as well as endoscopy and pathology reports—were assessed for histologic remission (<15 eos/hpf), complaint of dysphagia, esophageal food impaction, esophageal stricture occurrence, and the need for esophageal dilation that temporally corresponded to the second EGD of each treatment period.

The Wilcoxon rank-sum test was used to compare MPR values between treatment periods in which histologic remission had been achieved and those in which it had not. Youden’s index, in conjunction with receiver operating characteristic (ROC) analysis, was then employed to identify the optimum cut-point of MPR with respect to the achievement of histologic remission. The MPRs were then categorized as a binary variable representing the optimal cut-point for histologic remission. Lastly, odds ratios were calculated to characterize the relationship between the MPR and the EoE outcomes of dysphagia, food impaction, stricture occurrence, and the need for esophageal dilation; this was a multivariable model that included MPR status as an independent variable along with the covariates of age, sex, and race.

SAS 9.4 (SAS Institute, Cary, NC, USA) was used for all statistical analyses [26]. A *p* value < 0.05 was considered statistically significant. GraphPad Prism (GraphPad Software, Version 10.1.2, San Diego, CA, USA) was used to visually depict the analyzed data [27]. The Walter Reed National Military Medical Center Institutional Review Board reviewed this study and deemed it exempt. All authors had access to the study data, and reviewed and approved the final manuscript.

## 3. Results

The study cohort included 354 adult patients with EoE, from which 275 individual treatment periods were analyzed (Figure 1); the median age associated with each treatment period was 43 years (IQR 34–53), 225 (82%) patients were male, and 194 (71%) were white.

With any treatment, the median MPR in the histologic remission treatment period group was 0.91 (interquartile range [IQR] 0.63–1) vs. 0.63 (IQR 0.31–0.95) for the non-remission treatment period group (*p* < 0.001). Similarly, the median MPR for the treatment period groups without EoE symptoms and disease-related complications was higher when compared to the median MPR for the treatment period groups with EoE symptoms and disease-related complications: 0.9 (IQR 0.65–1) vs. 0.59 (IQR 0.28–0.93) for dysphagia, 0.80 (IQR 0.49–1) vs. 0.29 (IQR 0.15–0.83) for food impaction, 0.78 (IQR 0.46–1) vs. 0.59 (IQR 0.28–0.92) for stricture occurrence, and 0.8 (IQR 0.5–1) vs. 0.46 (IQR 0.27–0.93) for esophageal dilation; these findings were statistically significant in all cases (*p* < 0.05) (Table 1).

With PPI treatment alone, the median MPR in the histologic remission treatment period group was 0.88 (IQR 0.59–1) vs. 0.61 (IQR 0.31–0.92) for the non-remission treatment period group (*p* = 0.001). The median MPR for the treatment period groups without EoE symptoms and disease-related complications was higher when compared to the median MPR for the treatment period groups with EoE symptoms and disease-related complications, but not always statistically significant: 0.85 (IQR 0.57–1) vs. 0.59 (IQR 0.28–0.92) for dysphagia (*p* = 0.001), 0.76 (IQR 0.45–1) vs. 0.34 (IQR 0.17–0.74) for food impaction (*p* = 0.001), 0.72 (IQR 0.41–1) vs. 0.65 (IQR 0.31–0.93) for stricture occurrence (*p* = 0.196), and 0.74 (IQR 0.45–1) vs. 0.46 (IQR 0.28–0.94) for esophageal dilation (*p* = 0.044) (Table 1).

With TS treatment alone, the median MPR in the histologic remission treatment period group was 0.98 (IQR 0.88–1) vs. 0.69 (IQR 0.44–0.98) for the non-remission treatment period group (*p* = 0.02). The median MPR for the treatment period groups without EoE symptoms and disease-related complications was higher when compared to the median MPR for the treatment period groups with EoE symptoms and disease-related complications, but not always statistically significant: 0.95 (IQR 0.83–1) vs. 0.59 (IQR 0.27–0.92) for dysphagia (*p* = 0.005), 0.85 (IQR 0.61–1) vs. 0.14 (IQR 0.08–0.59) for food impaction (*p* = 0.071), 0.91 (IQR 0.63–1) vs. 0.58 (IQR 0.24–0.86) for stricture occurrence (*p* = 0.065), and 0.89 (IQR 0.63–1) vs. 0.48 (IQR 0.27–0.59) for esophageal dilation (*p* = 0.052) (Table 1).

With combination therapy (PPI + TS), the median MPR in the histologic remission treatment period group was 0.88 (IQR 0.63–1) vs. 0.63 (IQR 0.21–1) for the non-remission treatment period group (*p* = 0.08). The median MPR for the treatment period groups without EoE symptoms and disease-related complications was higher when compared to the median MPR for the treatment period groups with EoE symptoms and disease-related complications, but not always statistically significant: 0.88 (IQR 0.63–1) vs. 0.6 (IQR 0.26–0.94) for dysphagia (*p* = 0.09), 0.83 (IQR 0.58–1) vs. 0.19 (IQR 0.16–1) for food impaction (*p* = 0.156), 0.87 (IQR 0.6–1) vs. 0.51 (IQR 0.13–0.76) for stricture occurrence (*p* = 0.076), and 0.9 (IQR 0.6–1) vs. 0.44 (IQR 0.14–0.56) for esophageal dilation (*p* = 0.013) (Table 1).

**Figure 4:**
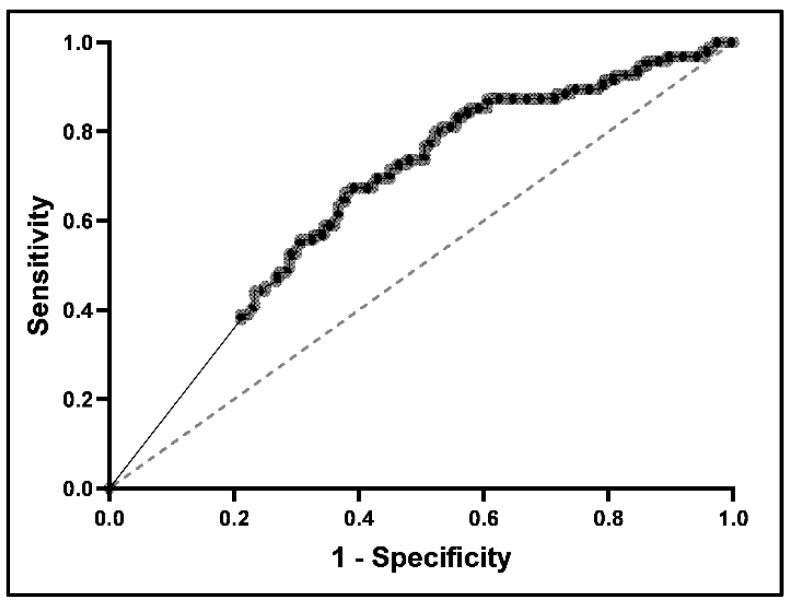
Receiver operator characteristic (ROC) curve of medication possession ratio and histologic remission for any treatment. The dark, dotted, curved line represents the ROC curve for our data, and the light, dashed, straight line represents the ROC curve for random guess with no predictive value.

**Figure 5:**
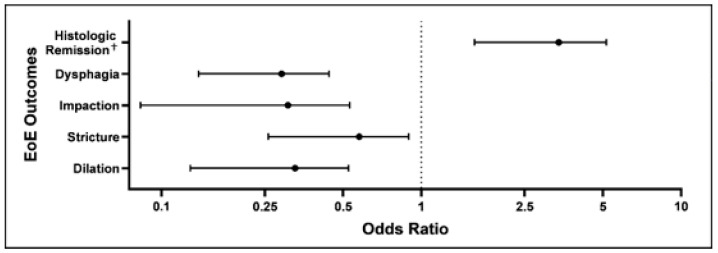
Odds ratios for the medication possession ratio ≥ 0.7 vs. medication possession ratio < 0.7. ^†^ <15 eosinophils per high-power field on esophageal biopsy.

For any treatment, the optimal MPR that correlated with histologic remission was 0.7 (Sen 0.66, Spec 0.62, AUC 0.63) according to Youden’s index in conjunction with receiver operating characteristic analysis (Figure 4). With MPRs ≥ 0.7, there were significantly increased odds of histologic remission (odds ratio 3.05, 95% confidence interval 1.79–5.30), and significantly decreased odds of dysphagia (OR 0.27, 95% CI 0.15–0.45), food impaction (OR 0.26, 95% CI 0.11–0.55), stricture occurrence (OR 0.52 95% CI 0.29–0.92), and esophageal dilation (OR 0.29, 95% CI 0.15–0.54) (Figure 5). As the amalgamated “any treatment” treatment period group had the highest power, ROC analysis was not performed for each specific treatment group.

## 4. Discussion

This is the first retrospective cohort study to assess the treatment adherence metric of MPR in adults with EoE. As such, we are the first to demonstrate that a higher MPR is significantly associated with improved histologic remission and symptom outcomes in EoE. Specifically, an MPR ≥ 0.7 (i.e., possessing the medicine for at least 70% of the duration of the treatment period) is associated with histologic remission and decreased odds of dysphagia, impaction, stricture, and dilation. Our results support a real-world patient counseling strategy that stresses how improved treatment adherence may lead to improved clinical outcomes. Such a strategy might include prospective adherence monitoring to improve overall adherence to treatment over time, even when the occasional dose is missed.

EoE is a challenging disease to manage because its disease activity can be difficult to assess using symptom reporting alone, often requiring follow-up endoscopic biopsy. This can make EoE disease monitoring costly. In 2015, it was shown that the median cost associated with EoE in the United States is USD 2302 per year per patient, with estimated total costs attributable to EoE ranging from USD 350 to USD 947 million per year [28]. A large portion of that cost is generated by EGDs. According to the U.S. Centers for Medicare & Medicaid Services, the cost of an EGD in 2021 averaged USD 549–949 nationally [29]. Additionally, while largely safe and well tolerated, patients receiving EGDs may experience the rare complications of hemorrhage and/or perforation, potentially necessitating hospitalization [20]. Independent of the procedure itself, there are also potential risks related to the anesthesia required to perform an EGD [30,31]. These risks are particularly relevant in pediatrics, as evidenced by the U.S. Food and Drug Administration “Drug Safety Communication” issued in December 2016, warning that the repeated use of anesthetics “may affect the development of children’s brains” [32]. Thus, decreasing EGDs in patients with EoE has the potential to substantially drive down cost, while also minimizing procedural risks.

As MPR may be considered a surrogate marker of adherence in the medical treatment of EoE, the calculation of a MPR before performing a repeat EGD may be able to decrease the performance of unnecessary EGDs for monitoring EoE activity. Specifically, based on our data, it may be practical to delay repeat EGDs for patients in whom the MPR is <0.7 and, also, lack other clinical indications for endoscopy. Furthermore, MPR may also have utility in understanding biopsy results that show a lack of histologic remission, as calculating a MPR may help differentiate disease activity caused by a lack of adherence to medication from true treatment refractory disease; this could spare unnecessary dose escalation or the modification of treatment in favor of re-doubling emphasis on medication adherence.

There have been calls in the gastroenterology community for the increased implementation of research findings and other evidence-based practices into routine practice, noting that optimizing treatment approaches, monitoring, and follow-up are key to improving the quality and effectiveness of the care provided by gastroenterologists [4,33]. Our innovative findings answer this call. Calculating a MPR for patients with EoE undergoing medical therapy has potential implications for clinical practice, as it may be a useful tool for identifying patients who are at risk of poor outcomes and who may benefit from precise interventions to improve medication adherence. Leveraging the use of health information technology to promote medication adherence has shown promising results in chronic diseases in which relationships between MPR and disease outcomes have been proven [34,35,36]. This might suggest that targeted, health-information-technology-based interventions to support medication adherence in patients with EoE could have a beneficial effect on EoE disease outcomes as well. Specifically, analyzing pharmacy use data may be a future target for quality improvement interventions in this patient population.

The limitations of this study include a relatively homogenous sample with respect to biologic sex and race, the inability to account for confounding variable such as disease severity, inter-provider inconsistencies in the dosing of PPIs, TSs, and combination therapy, as well as not accounting for any dosing changes that may have occurred during the period of time for which MPRs were calculated. Our study was also limited in that we did not assess outcomes other than histologic remission, dysphagia, food impaction, stricture occurrence, and esophageal dilation. Additionally, we could not exclude the possibility that patients were taking PPIs or TSs that were obtained over the counter, although we feel that this scenario is unlikely given our specific healthcare system, in which medications are dispensed free of charge. Further limiting generalizability, this study reviewed a time period, 2005–2020, before biologic medications for the treatment of EoE became widely available for routine clinical use [8,9,10]. There are also certain limitations to the use of MPR itself, such as the possibility of overestimating adherence [37]. While we are confident in our results, further studies of the correlation between MPR and EoE outcomes would be beneficial to the field of gastroenterology. Further studies might be improved by recruiting a larger and more diverse study population, standardizing the dosing of PPIs and TSs, including patients on biologic therapy approved for EoE, having a single pathologist (or group of pathologists) interpret the biopsies, and incorporating a prospective design with randomization, blinding, and accounting for potentially confounding variables. The incorporation of cost-effectiveness models might also be beneficial in further analyses.

## 5. Conclusions

In patients with EoE, maintenance therapy is necessary to achieve histologic remission and halt the development of fibrostenotic features. An efficient, real-world clinical strategy to quickly assess an EoE patient’s adherence to medical treatment is the calculation of a MPR. As we have shown here, a MPR ≥ 0.7 is associated with histologic remission, and with decreased odds of dysphagia, food impaction, stricture occurrence, and the need for esophageal dilation in patients with EoE. Our study answers the call of the gastroenterology community for the increased implementation of research findings and other evidence-based practices into routine practice. Calculating a MPR before performing a repeat EGD to assess a patient with EoE’s response to medical treatment may decrease unnecessary procedures, which would positively affect both patient safety and the cost of care.

## Figures and Tables

**Table 1:** Median medication possession ratio for treatment periods and EoE outcomes of histologic remission, dysphagia, food impaction, presence of stricture and need for dilation.

	Histologic Remission ^†^			Dysphagia			Impaction			Stricture			Dilation		
	YES	NO	*p* Value	NO	YES	*p* Value	NO	YES	*p* Value	NO	YES	*p* Value	NO	YES	*p* Value
**Any Treatment** **(n = 275)**	**95** (35%)	**180** (65%)		**112** (41%)	**163** (59%)		**239** (87%)	**36** (13%)		**209** (76%)	**66** (24%)		**216** (79%)	**59** (22%)	
**Medication Possession Ratio, median (IQR ^‡^)**	**0.91** (0.63–1)	**0.63** (0.31–0.95)	**<0.001**	**0.9** (0.65–1)	**0.59** (0.28–0.93)	**<0.001**	**0.8** (0.49–1)	**0.29** (0.15–0.83)	**<0.001**	**0.78** (0.46–1)	**0.59** (0.28–0.92)	**0.011**	**0.8** (0.5–1)	**0.46** (0.27–0.93)	**<0.001**
**Proton Pump Inhibitor** **(n = 188)**	**65** (35%)	**123** (65%)		**69** (37%)	**119** (63%)		**162** (86%)	**26** (14%)		**137** (73%)	**51** (27%)		**143** (76%)	**45** (24%)	
**Medication Possession Ratio, median (IQR ^‡^)**	**0.88** (0.59–1)	**0.61** (0.31–0.92)	**0.001**	**0.85** (0.57–1)	**0.59** (0.28–0.92)	**0.001**	**0.76** (0.45–1)	**0.34** (0.17–0.74)	**0.001**	**0.72** (0.41–1)	**0.65** (0.31–0.93)	**0.196**	**0.74** (0.45–1)	**0.46** (0.28–0.94)	**0.044**
**Topical Steroid (n = 40)**	**12** (30%)	**28** (70%)		**19** (48.5%)	**21** (52.5%)		**36** (90%)	**4** (10%)		**33** (82.5%)	**7** (17.5%)		**34** (85%)	**6** (15%)	
**Medication Possession Ratio, median (IQR ^‡^)**	**0.98** (0.88–1)	**0.69** (0.44–0.98)	**0.02**	**0.95** (0.83–1)	**0.59** (0.27–0.92)	**0.005**	**0.85** (0.61–1)	**0.14** (0.08–0.59)	**0.071**	**0.91** (0.63–1)	**0.58** (0.24–0.86)	**0.065**	**0.89** (0.63–1)	**0.48** (0.27–0.59)	**0.052**
**Combination Therapy** **(n = 47)**	**18** (38%)	**29** (62%)		**24** (51%)	**23** (49%)		**41** (87%)	**6** (13%)		**39** (83%)	**8** (17%)		**39** (83%)	**8** (17%)	
**Medication Possession Ratio, median (IQR ^‡^)**	**0.88** (0.63–1)	**0.63** (0.21–1)	**0.08**	**0.88** (0.63–1)	**0.6** (0.26–0.94)	**0.09**	**0.83** (0.58–1)	**0.19** (0.16–1)	**0.156**	**0.87** (0.6–1)	**0.51** (0.13–0.76)	**0.076**	**0.9** (0.6–1)	**0.44** (0.14–0.56)	**0.013**

^†^ <15 eosinophils per high-power field on esophageal biopsy; ^‡^ Interquartile range.

## Data Availability

The data presented in this study are available upon request from the corresponding author.
